# Invasive alien plants in riparian ecosystems: a study from two lowland rivers in Eastern Poland

**DOI:** 10.1007/s11356-026-38279-6

**Published:** 2026-10-07

**Authors:** Joanna Sender, Monika Różańska-Boczula

**Affiliations:** 1 Institute of Soil Science, Environment Engineering and Management, University of Life Sciences in Lublin, Str. Leszczyńskiego 7, 20-950 Lublin, Poland; 2 Department of Applied Mathematics and Computer Science, University of Life Sciences in Lublin, Str. Akademicka 13, 20-950 Lublin, Poland

**Keywords:** Riparian ecosystems, Floristic diversity, Invasive alien species, Habitat characteristics, Ellenberg indicator values, Index of Biotic Integrity, Community ordination

## Abstract

Riparian ecosystems are biodiversity hotspots but are also highly vulnerable to biological invasions due to strong hydrological connectivity, recurrent disturbance and exposure to nutrient inputs. This study examined patterns of invasive alien plant species (IAS) occurrence along two lowland rivers in eastern Poland (the Biala Lada and Czarna Lada). Vegetation was surveyed at ten sites representing different land-use contexts using 150 phytosociological plots. Floristic diversity metrics, Ellenberg indicator values (EIVs), and a vegetation-based Index of Biotic Integrity (IBI) were used to characterise vegetation composition and ecological conditions. Logistic regression based on species-level Ellenberg indicator classes showed that invasive species differed significantly from non-invasive taxa in their ecological preferences. Species belonging to higher nitrogen and temperature indicator classes were more likely to be classified as invasive, indicating a stronger representation of eutrophic and thermophilous taxa within the invasive species pool. In contrast, soil reaction showed only a marginal relationship with invasion status. Sites with greater IAS occurrence were characterised by reduced evenness, increased dominance of ruderal taxa and stronger floristic homogenisation. The vegetation-based Index of Biotic Integrity (IBI) reflected variation in ecological quality across the studied riparian sites. The results suggest that invasive species in the studied river valleys are associated with distinct ecological preference profiles, particularly with respect to nutrient availability and temperature requirements. Field-based Ellenberg indicators and the vegetation-based Index of Biotic Integrity (IBI) describe variation in habitat conditions among the studied sites, while the species-level Ellenberg classes describe the ecological attributes of the local invasive versus non-invasive species pool; together these indicators provide complementary tools for characterising riparian vegetation and invasion-related patterns.

**Supplementary Information:**

The online version contains supplementary material available at https://doi.org/10.1007/s11356-026-38279-6.

## Introduction

Riparian ecosystems are widely recognised as biodiversity hotspots and, at the same time, as some of the most invasion-prone habitats worldwide. Their linear structure, high connectivity and recurrent disturbances create efficient dispersal pathways for invasive alien plant species (IAS), facilitating transport both longitudinally and laterally along river networks (Van Oorschot et al. 2017; Wagner et al. 2020). IAS can reduce native species richness, alter vegetation structure and promote floristic homogenisation, whereby disturbance-tolerant taxa replace locally characteristic assemblages (Aronson et al. 2014; Olden and Rooney 2006). This process of homogenisation is increasingly recognised as a consequence of both species invasiveness and community invasibility, especially in riparian corridors where ecological filters are weakened (Richardson and Pyšek 2006). These changes undermine ecosystem functioning and resilience, particularly where nutrient inputs, hydrological alteration and landscape fragmentation amplify invasion pressure (Hobbs and Huenneke 1992; Vilà et al. 2011).

Across Europe, plant invasions in riparian zones are strongly associated with land-use intensity, nutrient enrichment and landscape connectivity. Studies from Germany, the Czech Republic and the Baltic region show that eutrophication and recurrent disturbance favour disturbance-tolerant and fast-growing invaders such as *Impatiens glandulifera* and *Solidago gigantea*, while fragmented riparian woodlands facilitate the establishment of woody alien taxa by altering vegetation structure and resource availability (Chytrý et al. 2005; Hejda et al. 2009; Gudžinskas et al. 2020).

Similar patterns have been reported from the Danube Basin and the Iberian Peninsula, where propagule pressure and hydromorphological modification enhance colonisation success (Anđelković et al. 2022; Castro-Díez and Alonso 2017). These findings collectively underline the continental scale of riparian invasions and the need for robust ecological indicators to detect vulnerable habitats. At the same time, vegetation-based assessments require integration with broader landscape and policy frameworks, as highlighted by Dufour et al. (2019) in their analysis of emerging priorities in riparian research.

Vegetation-based indicators offer practical tools for describing variation in habitat condition and linking environmental filters with observed patterns of invasive species occurrence. Ellenberg indicator values (EIVs) are widely used proxies for resource availability and disturbance regimes, providing practical assessments of nutrient status, moisture, light and soil reaction (Schaffers and Sýkora 2000; Diekmann 2003). Composite indices such as the Index of Biotic Integrity (IBI) integrate multiple dimensions of ecosystem condition, enabling comparisons across habitat types and gradients of anthropogenic pressure (Karr and Chu 1999). Both indicators are primarily descriptive rather than inferential tools, which makes them well suited to detecting broad ecological patterns in exploratory, site-based studies.

Despite extensive research across Europe, lowland river valleys in Central–Eastern Europe remain under-represented in invasion ecology. To address this gap, we examined floristic diversity, vegetation integrity, and ecological characteristics associated with invasive alien plant species along two lowland rivers in eastern Poland. Specifically, we asked (1) whether invasive and non-invasive species differ in their ecological characteristics as expressed by Ellenberg indicator values; (2) how patterns of species diversity and floristic homogenisation vary along gradients of invasion species occurrence; and (3) whether a vegetation-based IBI can serve as a practical indicator of ecological conditions associated with invasive alien species occurrence. The study is therefore framed as an exploratory case study aimed at documenting local ecological patterns rather than deriving broadly generalisable conclusions.

## Materials and methods

### Study area and site selection

The study was conducted in two lowland river valleys in eastern Poland: the Biala Lada and the Czarna Lada, located within agricultural and semi-natural landscapes typical for Central–Eastern Europe. Both rivers are subject to increasing anthropogenic pressure, including channel modification, urbanisation and land-use intensification, which influence riparian vegetation and the establishment of invasive alien plant species. The studied rivers are lowland tributaries of the Tanew River within the Vistula River basin. They represent small to medium-sized watercourses typical of the region, with relatively low discharge and predominantly agricultural catchments. River parameters at the analysed sections are included in Supplementary Table S1.

Ten study sites (five along each river) were selected to represent the dominant land-use contexts in the valleys. Six land-use categories were distinguished: allotment gardens, hydrotechnical infrastructure, residential/built-up areas, tree plantations, semi-natural meadows, and agricultural land. Some categories were represented by a single site (e.g. allotment gardens and agricultural land), and were included to capture the range of habitat conditions present in the study area rather than for direct comparison among land-use types. Hydrotechnical infrastructure sites included river sections modified by engineering structures such as bridges, reinforced banks and channel regulation works, resulting in altered channel morphology and vegetation structure.

**Fig. 1:**
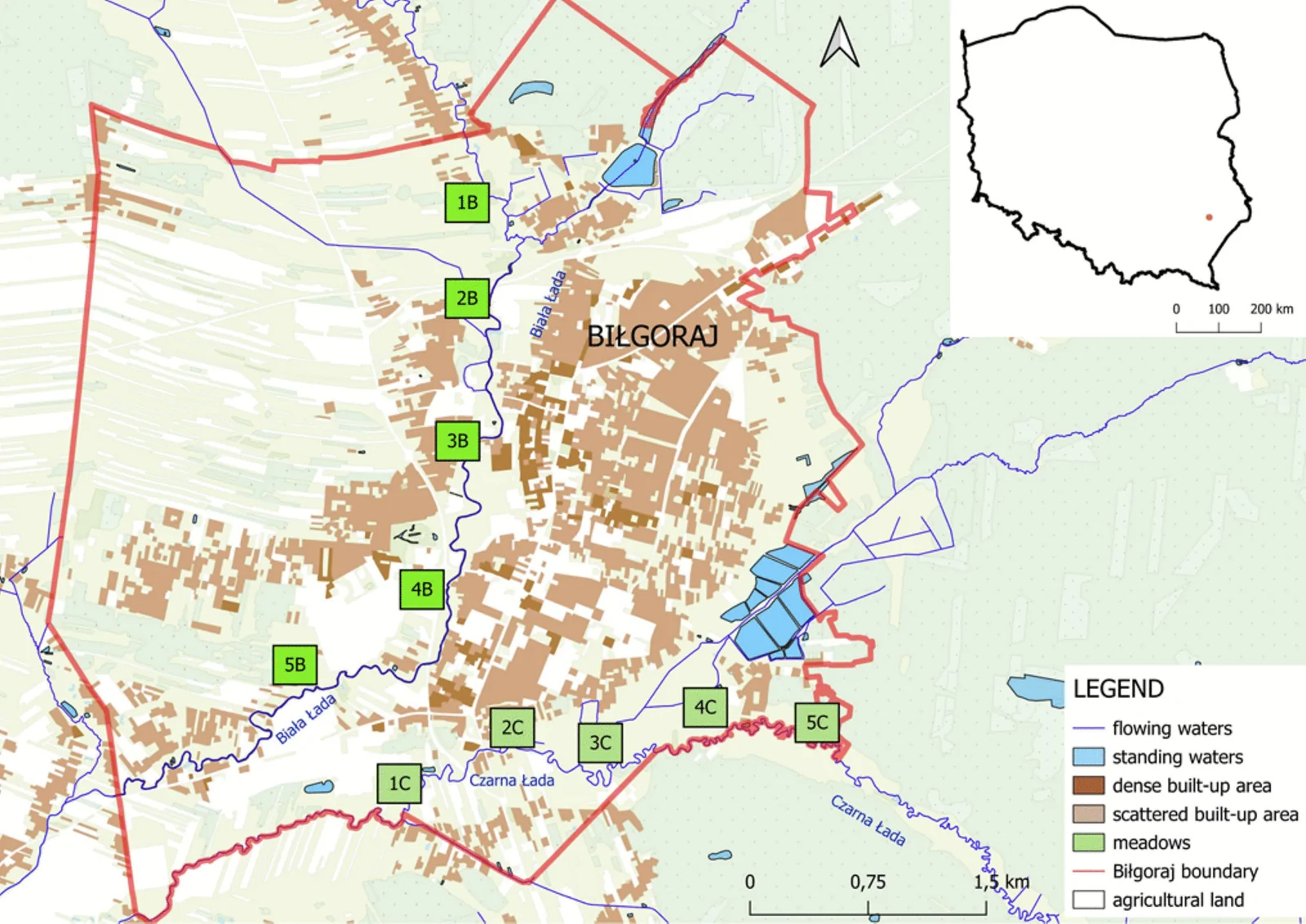
Location of the study area in eastern Poland and distribution of the ten sampling sites along the Biala Lada (1B–5B) and Czarna Lada (B) river valleys. Land-use categories include allotment gardens (1B), hydrotechnical infrastructure (2B, 2 C), residential areas (3B, 3 C), tree plantations (4B, 4 C), meadows (5B, 5 C), and agricultural land (1C)

Land-use categories were used to structure site selection and to provide descriptive context, but were not treated as independent factors in statistical analyses due to limited and uneven representation of land-use categories, with some categories represented by a single site. Site locations and land-use categories are shown in Fig. 1.

### Vegetation sampling

Field surveys were carried out between spring 2023 and spring 2025. Vegetation was recorded using phytosociological plots (quadrats) of 2 × 2 m, following standard practice for riparian herbaceous and shrub vegetation. At each of the ten study sites, fifteen plots were surveyed, yielding a total of 150 plots. Plots were arranged along linear transects placed perpendicular to the river channel in order to capture within-site variation along the river–valley gradient. At each site, five transects were established within the selected site area, reflecting the local land-use context and spatial configuration of the riparian zone. Transects were distributed within the riparian zone at variable distances (typically ca. 10–20 m apart), and their length was adjusted to the local width of the riparian zone (ca. 25–150 m). Each transect consisted of three plots located from the vicinity of the river channel towards the valley margin, representing the beginning, middle and end of the gradient. Transects served solely as a spatial framework for plot placement and were not treated as an independent sampling unit. Plots and transects were considered subsamples nested within sites. To improve clarity of the sampling design, a schematic representation of the spatial arrangement of transects and vegetation plots within a study site is provided in the Supplementary Fig. S1. The selected plot size and spatial arrangement were intended to characterise local habitat variation rather than to provide an exhaustive species inventory or full statistical representativeness of each site. Species–area curves were therefore not applied, as the primary objective was comparative analysis among sites under contrasting land-use and habitat conditions. Within each plot, species composition and cover of the herbaceous layer were recorded. Tree and shrub cover were assessed at the site-level based on the overall vegetation structure observed along transects, as these layers extended beyond individual plot boundaries. Hydrological conditions were not measured directly during field surveys and are therefore discussed only qualitatively based on observed habitat characteristics.

Vegetation was recorded using the Braun–Blanquet cover–abundance scale (classes + to 5), which is commonly applied in riparian vegetation studies (Wysocki and Sikorski 2009). Braun–Blanquet values were subsequently transformed into cover-weighted data and used as input for diversity indices, Ellenberg indicator value calculations, ordination analyses and the vegetation-based Index of Biotic Integrity.

Special attention was given to alien, naturalised, invasive and synanthropic plant taxa. Species status refers here exclusively to biogeographical and invasion-related categories, following national and international classifications (Zając and Zając 2001; Mirek et al. 2002; Tokarska-Guzik 2005; Tokarska-Guzik et al. 2012; IUCN Invasive Species Specialist Group 2024).

Alien species were defined as taxa introduced intentionally or unintentionally outside their natural distribution range. Naturalised alien species were defined as alien taxa forming self-sustaining populations without direct human assistance but not exhibiting rapid spread or documented negative ecological impacts. IAS were defined as a subset of alien taxa capable of rapid spread and causing negative ecological or socio-economic impacts. Synanthropic species were defined as taxa closely associated with human-modified habitats, irrespective of their biogeographical origin. Generalist species were defined as taxa characterised by a broad ecological niche breadth and tolerance to a wide range of environmental conditions, allowing them to occur across multiple habitat types, including both semi-natural and human-modified riparian environments (Futuyma and Moreno 1988; Devictor et al. 2010). Within the studied river valleys, representative generalist species included *Urtica dioica*, *Poa trivialis*, *Plantago major* and *Taraxacum officinale* agg., which were recorded across multiple sites representing a range of habitat conditions. Plant assemblages were assigned to semi-natural, ruderal, synanthropic or anthropogenic categories through expert-based qualitative assessment integrating floristic composition, dominance structure and the presence of diagnostic indicator taxa, with indicator taxa given primary weight in ambiguous cases. Semi-natural assemblages were dominated by native species with limited signs of direct human disturbance; ruderal assemblages were characterised by disturbance-tolerant species associated with frequent physical or nutrient disturbance; synanthropic assemblages were dominated by species closely associated with human-managed environments; and anthropogenic assemblages reflected vegetation strongly shaped by direct land use or infrastructure. These categories were used descriptively to support the interpretation of vegetation structure and disturbance context across sites and were not included as independent variables in statistical analyses.

### Ecological indicator values (EIVs)

For each plot, ecological indicator values (EIVs) were calculated for light (L), temperature (T), moisture (F), soil reaction (R) and nitrogen (N). The values followed the Polish adaptation of Ellenberg’s system (Zarzycki et al. 2002), using cover-weighted means based on species abundances (Ellenberg et al. 1992). EIVs served as proxies for local site conditions and potential successional trends. The continentality index (K) was omitted because the study area is geographically uniform and does not exhibit meaningful variation along this gradient. Cover-weighted means were applied to reflect the contribution of individual species to plot-level environmental conditions. These cover-weighted, plot- and site-level EIVs (hereafter field-based EIVs) are conceptually distinct from the fixed, literature-based species-level Ellenberg indicator classes used in the species-level analysis in the “Ecological characteristics associated with invasive species occurrence” section, which represent an invariant ecological attribute of each taxon rather than a site-measured condition.

### Biodiversity assessment and Index of Biotic Integrity (IBI)

Species diversity was quantified using the Shannon–Wiener (*H*′) and Simpson (D) indices. In the Shannon–Wiener index, ${H}^{\prime}=-{\sum}_{i=1}^{s}{p}_{i}\cdot ln {p}_{i}$, where *s* is the number of species and ${p}_{i}$ is the proportion of individuals of species i in the total community. The Simpson index, $D=1-{\sum}_{i=1}^{s}{p}_{i}^{2}$, was used to complement Shannon–Wiener diversity, with higher values indicating greater diversity and lower dominance.

The indices were calculated at the plot level and averaged per site. To integrate multiple ecological dimensions, we constructed a vegetation-based Index of Biotic Integrity (IBI). The approach is based on the conceptual foundations of multimetric biological assessment (Karr 1981; Karr and Chu 1999), adapted here for riparian vegetation. The IBI incorporates species diversity (*H*′, *D*), the share of alien, invasive and synanthropic taxa, the presence of indicator species, and cover-weighted Ellenberg indicator values (EIVs) for moisture and nitrogen. Indicator species were defined as taxa characteristic of particular habitat types or vegetation units, whose presence and relative abundance indicate specific ecological conditions and disturbance regimes, qualitatively following established phytosociological concepts, rather than a formal statistical indicator species (IndVal) analysis. Cover-weighted EIVs were used as ecological proxies for moisture and nutrient conditions, following widely established applications in European vegetation studies (Schaffers and Sýkora 2000; Diekmann 2003).

The scoring system was developed with reference to ecological classification approaches used under the EU Water Framework Directive (Directive 2000/60/EC; European Parliament and Council 2000) and adapted to pilot-scale riparian vegetation surveys. Detailed scoring rules and thresholds are provided in the Supplementary Table S2, whereas ecological status classes are summarised in Supplementary Table S3.

### Statistical analyses

The study project included ten area units: five along the Biala Lada River and five along the Czarna Lada River. Each area unit represented a locally occurring type of land-use in the riparian zone, and five transects were designated within each unit. Transects were therefore treated as subsamples nested within area units rather than as independent replications of land-use categories. It should be noted, however, that several categories were represented by only two area units, and some spatial development contexts occurred only in one of the two rivers. As a result, formal conclusions on categorical land-use effects were limited because land-use effects could not be reliably separated from site-level and river-specific conditions. Therefore, land-use types were treated only as a descriptive context and land-use results were interpreted as local exploratory observations within the studied river valleys.

Species diversity was characterised using the Shannon–Wiener (*H*′) and Simpson (*D*) indices. Both indices were calculated for each plot and averaged at the site-level, providing descriptive measures of biodiversity across sites representing a range of habitat conditions.

Variation in species composition among sites was explored using non-metric multidimensional scaling (NMDS) based on Bray–Curtis dissimilarity, a widely applied ordination technique in vegetation ecology (Legendre and Legendre 2012). The analysis was performed using a species matrix restricted to invasive alien plant taxa in order to specifically assess patterns of IAS composition across sites. Ordination results were used to visualise the distribution of invasive alien species (IAS) across sites.

Principal component analysis (PCA) was used as an exploratory ordination to reduce the dimensionality of site-level data and to summarise multivariate similarities among study sites. Prior to PCA, variables were standardised (*z*-score transformation) to ensure comparability among variables measured on different scales. The analysis was based on a combined matrix of Ellenberg indicator values (L, T, F, R, N), vegetation structure descriptors (tree (TC%), shrub (SC%) and herbaceous (HV%) cover) and channel morphology variables (riverbed width and bank slope). To address potential artefacts arising from the use of community-weighted mean values, a permutation test was implemented following Zelený and Schaffers (2012). In this test, the variance explained by the first principal component was compared with a null distribution generated from 1000 random permutations, allowing an evaluation of whether the observed gradients differed from random expectations.

The ecological characteristics associated with invasion status were examined using an exploratory logistic regression framework. The analysis was conducted at the species level, using unique species as observational units. In this dataset, each row corresponded to one unique species (*n* = 217); the response variable was the species’ invasion status (1 = invasive, 0 = non-invasive), and the predictors were the species’ fixed Ellenberg indicator classes. Plots, transects and sites were not rows in this model and no nested random-effects structure was applied, since the unit of analysis was the species rather than a plot-level occurrence record. Each species was assigned a fixed set of Ellenberg indicator classes for nitrogen (Nc), temperature (Tc), soil reaction (Rc), moisture (Fc) and light (Lc), taken directly from the tabulated literature values of Zarzycki et al. (2002) (Supplementary Table S6). These classes are identical for a given species irrespective of the site at which it occurs, in contrast to the cover-weighted, field-based EIVs calculated per plot and site (Ecological indicator values (EIVs)” section). Species-specific Ellenberg indicator classes were considered as explanatory variables. Model performance was evaluated using Akaike’s Information Criterion (AIC), pseudo-*R*^2^ coefficients and the area under the curve (AUC) from receiver operating characteristic (ROC) analysis. Potential collinearity among the included variables was assessed using variance inflation factors (VIF). Logistic regression was used to identify species-level ecological characteristics associated with invasion status, whereas transect-level mixed-effects models were used to test whether proportional IAS cover was related to weighted mean Ellenberg indicator values.

All statistical analyses were performed in Python 3.13.2 (including numpy, pandas and scikit-bio for ordination procedures) and in Statistica 13.3 (TIBCO Software Inc., Palo Alto, USA) (Stanisz 2007).

## Results

### Species diversity and occurrence of alien and invasive plants

**Fig. 2:**
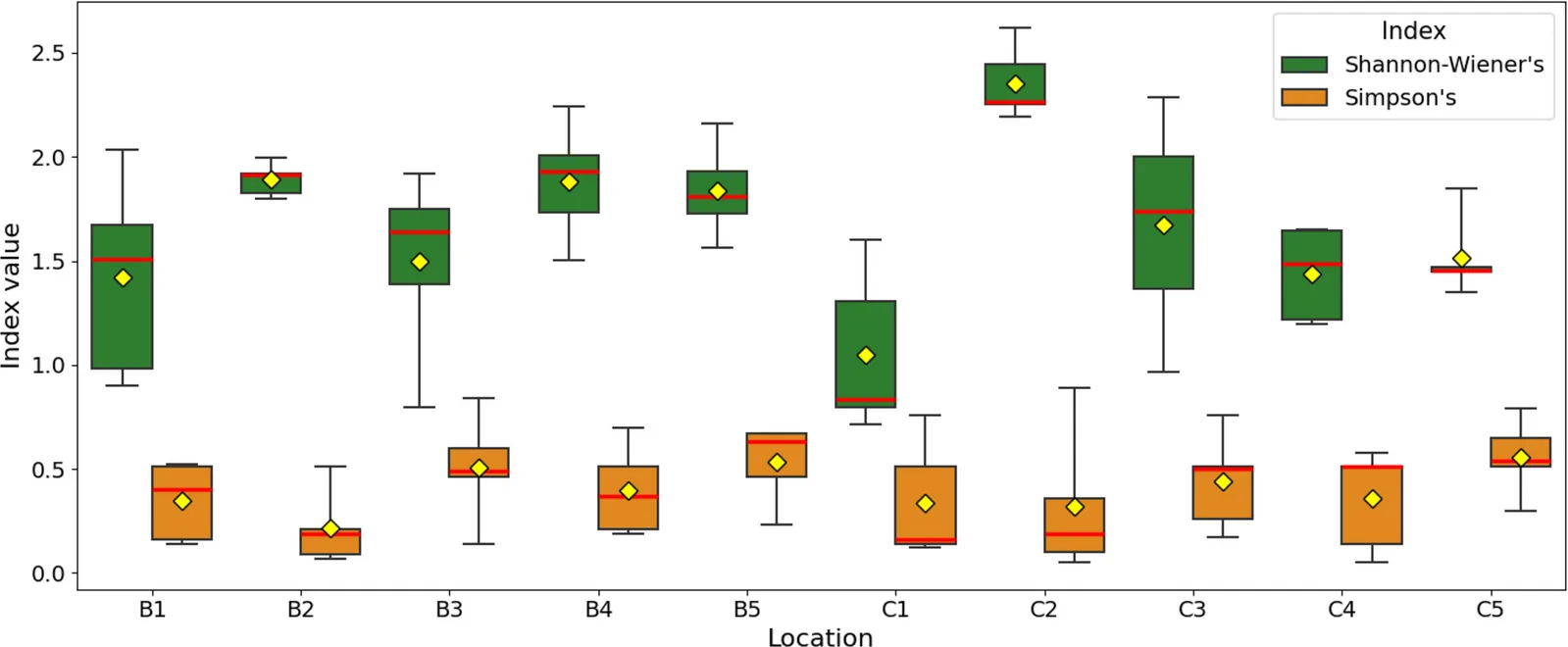
Site-level distributions of Shannon–Wiener diversity (*H*′) and Simpson’s diversity index (D) across the ten study sites (B1–B5, C1–C5). Shannon’s index (*H*′) and Simpson’s index (D) were calculated at the plot level and averaged per site. Higher values of both indices indicate greater diversity and lower dominance. Boxplots show the median (red horizontal line), the mean (yellow diamond), the interquartile range (box), and the minimum–maximum range (whiskers), based on 15 plots per site

In addition to overall floristic diversity, particular attention was given to the occurrence and distribution of invasive alien species (IAS) across sites. At the site-level (*n* = 10), Shannon’s diversity (*H*′) ranged from 2.4 to 3.4 (Fig. 2). The highest value was observed at site C2, while relatively high values were also recorded at B1 and C3–C4. Lower values occurred at sites such as B5 and C1. Simpson’s diversity index ranged from 0.13 to 0.82, with higher values indicating greater diversity and lower dominance. Along the Biala Lada, the highest Simpson diversity was recorded at B5, whereas the lowest was recorded at B2. Along the Czarna Lada, the highest Simpson diversity was recorded at C4, whereas the lowest was recorded at C1. Lower Simpson values therefore indicate stronger dominance by a few species. Site C4 showed relatively high values of both *H*′ and *D*, indicating a species-rich assemblage with a relatively even abundance distribution.

Across both valleys, several alien and invasive taxa were recorded in Supplementary Table S4. *Solidago gigantea* was the most frequent species, occurring at four sites representing both agricultural and built-up contexts (B1, B2, C1, C3). *Acer negundo* was recorded at two sites associated with residential or infrastructure settings (B3, C2), while *Robinia pseudoacacia* occurred sporadically near built-up areas. Overall, invasive taxa were more frequently observed in open, disturbed and nutrient-enriched riparian habitats, whereas their occurrence was lower in structurally more complex sites, as reflected by their distribution across sites (Supplementary Table S4).

### Differentiation of invasive species assemblages

**Fig. 3:**
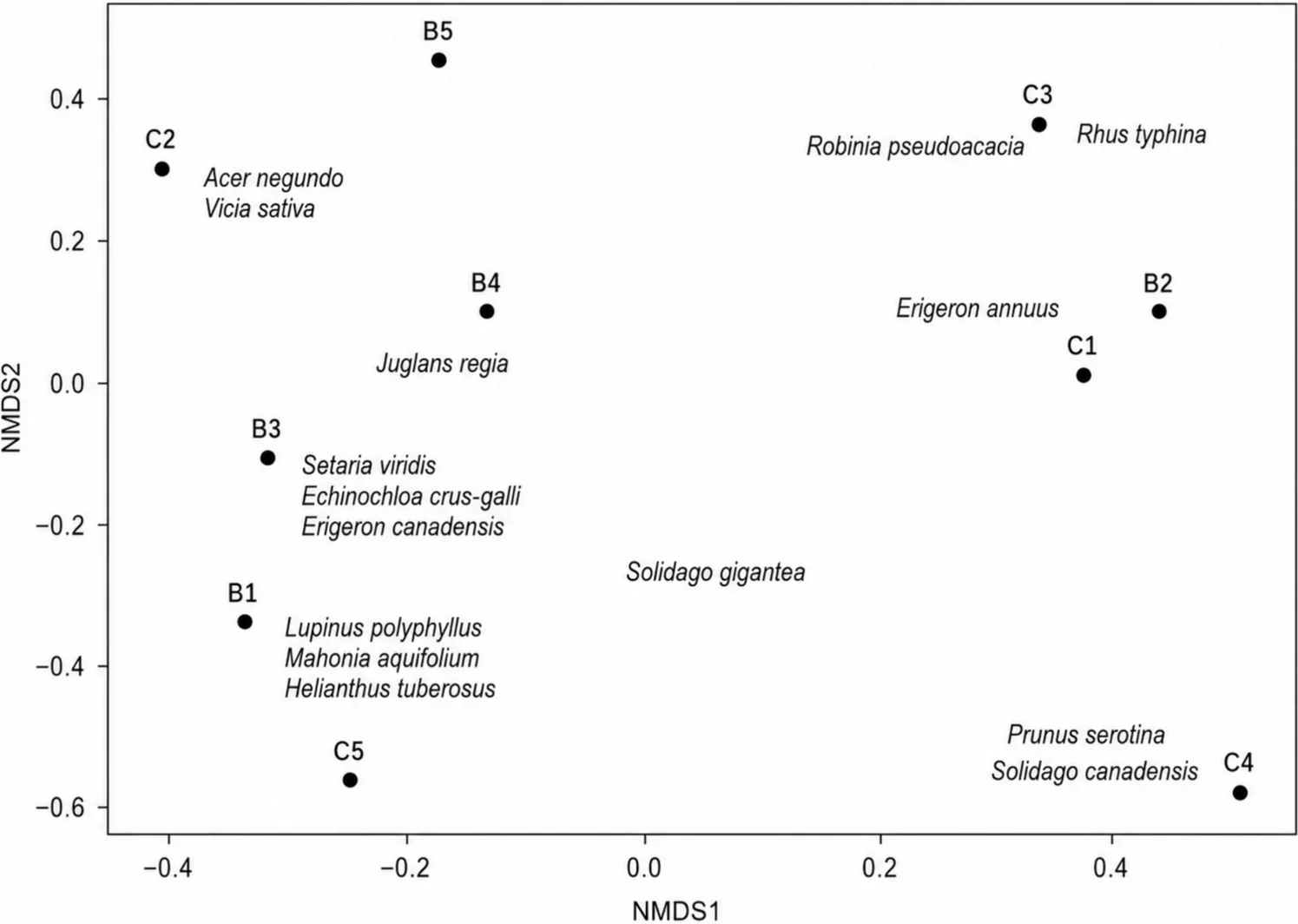
Non-metric multidimensional scaling (NMDS) ordination of invasive plant species assemblages at the ten study sites along the Biala Lada (B1–B5) and Czarna Lada (C1–C5), based on Bray–Curtis dissimilarity calculated from presence/absence data. Presence/absence was used to emphasise compositional differences among sites and to avoid disproportionate influence of highly dominant species arising from semi-quantitative Braun–Blanquet cover estimates

A site-level non-metric multidimensional scaling (NMDS) analysis based on the presence of invasive plant species illustrated differences between assemblages recorded along the Biala Lada (B1–B5) and Czarna Lada (C1–C5) (Fig. 3). The ordination was derived from a Bray–Curtis dissimilarity matrix and provided a two-dimensional solution (stress < 0.2). Sites along the Biala Lada clustered closely in ordination space, indicating a relatively homogeneous composition of invasive species. These assemblages were characterised by the frequent occurrence of herbaceous alien taxa such as *Erigeron canadensis*, *Echinochloa crus-galli*, *Helianthus tuberosus*, *Lupinus polyphyllus*, and *Mahonia aquifolium*. Residential (B3) and infrastructure-adjacent (B2) sites were positioned close to each other in the ordination space.

By contrast, sites along the Czarna Lada were more widely dispersed in the ordination space, indicating greater variation in invasive species composition. Woodland-associated sites (C3, C4) were associated with woody alien taxa such as *Rhus typhina*, *Robinia pseudoacacia*, *Prunus serotina* as well as tall perennial alien species including *Solidago canadensis*. Site C4 occupied a peripheral position in the ordination.

Infrastructure-related site C2 was associated with *Acer negundo* and *Vicia sativa.* Agricultural sites (B1, C1) occupied isolated positions in ordination space, while meadow sites (B5, C5) were located at the margins of the diagram.

### Habitat and environmental conditions

**Fig. 4:**
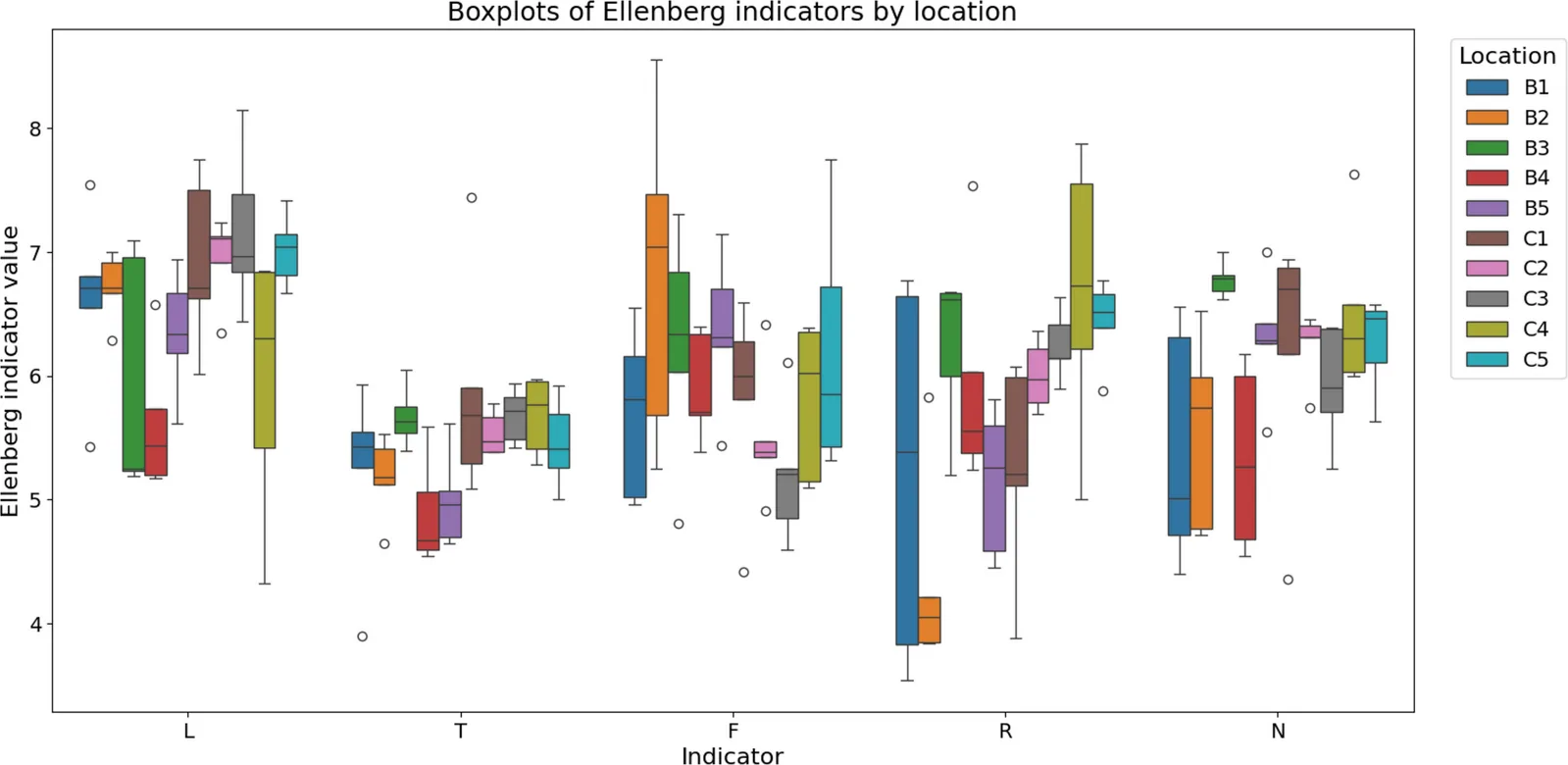
Boxplots showing the variability and distribution of Ellenberg indicator values (L, T, F, R, and N) across the ten study sites located along the Biala Lada (B1–B5) and Czarna Lada (C1–C5) rivers. Different colours represent individual study sites. The boxplots display the median (horizontal line), interquartile range (box), and the range of non-outlier values (whiskers) and circles represent outliers

Because land-use categories were weakly replicated, results involving land-use context are presented descriptively and are not interpreted as evidence of categorical land-use effects. Variation in Ellenberg indicator values (EIVs) derived from species composition was observed among sites (Fig. 4). Light indicator values (L) ranged from 5.7 to 7.0, with the highest values recorded at B5 (7.0) and C5 (6.9), and the lowest at B4 (5.7) and C4 (5.8). Moisture values (F) ranged from 4.4 to 5.4, with relatively higher values observed at some sites along the Czarna Lada (e.g. C5 (5.4), C4 (5.1)), whereas lower values occurred at sites such as B1 (4.5) and C1 (4.4).

Nitrogen indicator values (N) ranged from 5.1 to 6.5 and showed variation among sites, with relatively elevated values at B5 (6.5), B3 (6.1), C5 (6.3), and C3 (6.2), while lower values were recorded at C2 (5.1). Soil reaction values (R) ranged from 5.0 to 6.2 with the highest values recorded at B5 (6.2) and C5 (6.1), and the lowest at B1 (5.0) and C1 (5.2).

The boxplot distributions (Fig. 4) indicate considerable within-site variability for several indicators; therefore, the observed differences should be interpreted as descriptive patterns rather than consistent differences between river systems.

### Riparian habitat differentiation based on Ellenberg values and vegetation structure

**Fig. 5:**
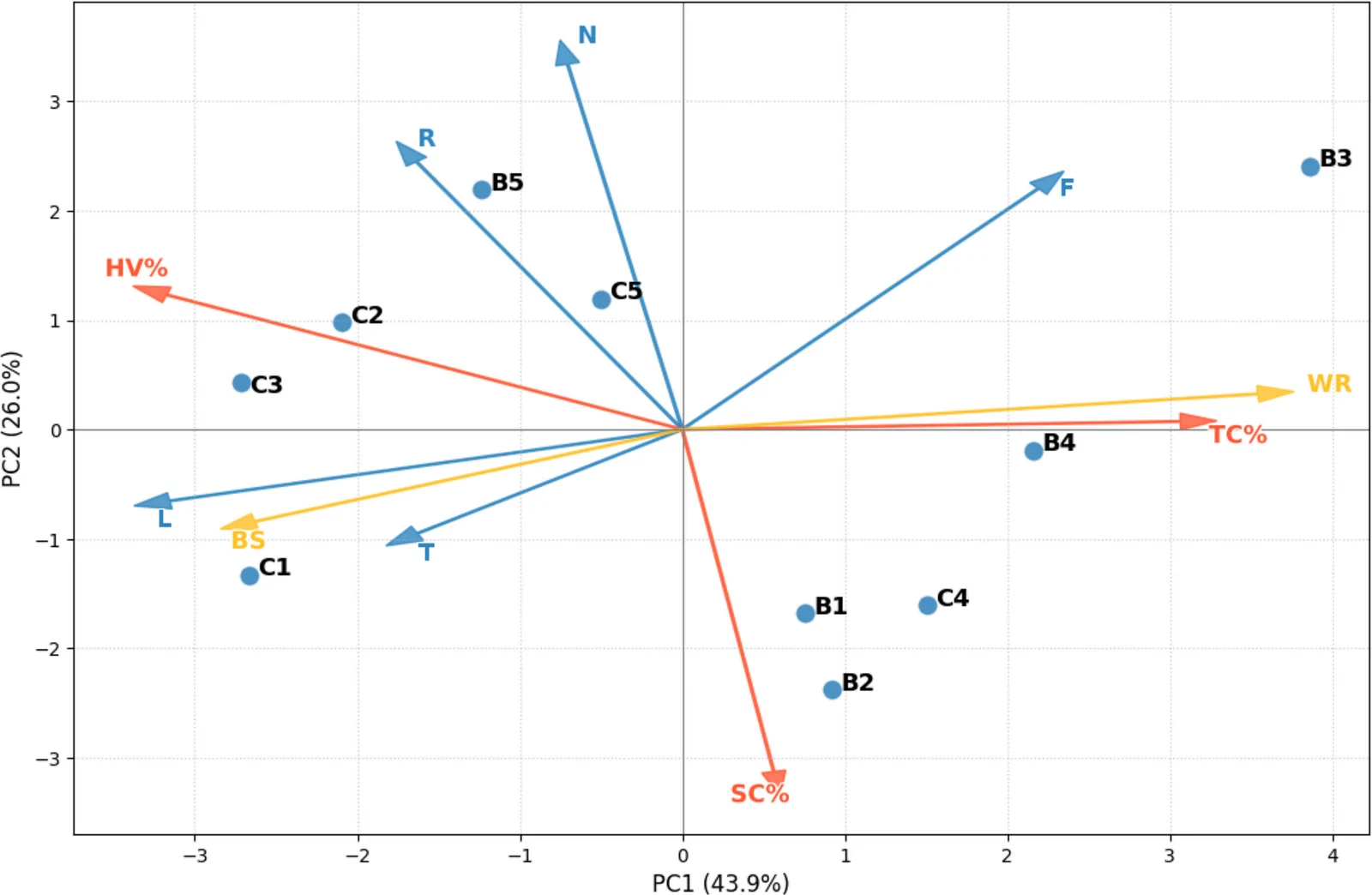
Principal component analysis (PCA) biplot illustrating multivariate similarities among the ten study sites (points; B1–B5, C1–C5) based on Ellenberg indicator values (L—light, T—temperature, F—moisture, R—soil reaction, N—nitrogen), vegetation structure descriptors (tree cover—TC%, shrub cover—SC%, herbaceous cover—HV%) and channel morphology variables (bank slope—BS, channel width—WR). Points represent site scores, while arrows indicate variable loadings, with arrow length reflecting the strength of association with the ordination axes. Colours distinguish variable groups: blue—Ellenberg indicator values, orange—vegetation structure variables, yellow—channel morphology variables. The ordination is exploratory and intended to summarise integrated site-level gradients rather than to infer causal relationships

A principal component analysis (PCA) was applied as an exploratory ordination to summarise multivariate similarities and contrasts among the study sites based on a combined set of Ellenberg indicator values (L, T, F, R, N), vegetation structure descriptors (tree, shrub and herbaceous cover) and channel morphology variables (riverbed width and bank slope) (Fig. 5). The analysis was intended to provide a descriptive synthesis of integrated site characteristics rather than to explain vegetation patterns as a function of environmental variables.

The first two axes together accounted for 69.9% of the variance (PC1 = 43.9%, PC2 = 26.0%; Supplementary Table S5). A permutation test indicated that the structure of PC1 was unlikely to be an artefact of Ellenberg indicator averaging, supporting the interpretation of non-random site-level structure. PC1 contrasted sites characterised by wider channels and higher tree cover (positive scores) with steeper, more open sites dominated by herbaceous vegetation and higher light availability (negative scores). This axis therefore summarised variation from more structurally complex, shaded riparian sections to more open and morphologically constrained sites. PC2 separated sites associated with higher nutrient availability and base-rich conditions (positive correlations with nitrogen and soil reaction) from sites characterised by greater shrub cover (negative scores). This axis summarised co-variation between edaphic proxies and woody understorey development across the studied sites. PC3 explained a further 14.5% of the variance and showed a moderate association with temperature, but did not represent an interpretable additional pattern and was therefore not considered further.

This ordination summarised variation among sites (site scores) in functional composition and vegetation structure, as jointly captured by the Ellenberg indicator values and vegetation structure variables entered into the analysis, and was used as an exploratory visualisation tool. Residential sites (B3, C3) were positioned at high PC1 scores, corresponding to wider channels and higher tree cover. The allotment site (B1) and agricultural site (C1) scored negatively along PC1, reflecting steeper banks, open light conditions and herbaceous dominance. Meadow sites (B5, C5) occupied high PC2 positions, consistent with nutrient-enriched, base-rich conditions and limited shrub development. Bridge-associated sites (B2, C2) scored negatively on PC2, indicating stronger shrub encroachment and lower nitrogen and soil reaction values. Woodland sites (B4, C4) occupied intermediate positions in the ordination space, reflecting mixed structural and edaphic characteristics.

Overall, the PCA ordination summarised multivariate variation among sites, reflecting co-variation between Ellenberg indicator values, vegetation structure and channel morphology variables.

### Ecological characteristics associated with invasive species occurrence

To identify ecological characteristics associated with invasion status, an exploratory logistic regression model was applied using unique species as observational units (*n* = 217 species). Species invasion status (invasive = 1, non-invasive = 0) was used as the dependent variable, whereas species-level Ellenberg indicator value classes (Lc, Fc, Rc, Nc and Tc—ecological classes defined in Supplementary Table S6) were included as explanatory variables. These are fixed, literature-based values assigned to each taxon (Zarzycki et al. 2002), identical for a given species regardless of site, and therefore distinct from the cover-weighted, field-based EIVs used elsewhere in this study (“Ecological indicator values (EIVs)”, “Habitat and environmental conditions”, and “Riparian habitat differentiation based on Ellenberg values and vegetation structure” sections). Thus, the analysis evaluated differences in the literature-based ecological preferences of species classified as invasive versus non-invasive within the regional species pool, rather than differences in habitat conditions actually measured at the study sites. Variance inflation factors (VIF < 2) indicated no problematic collinearity among predictors. The fitted model was:

$$logit\ P=-8.10-0.74\cdot {R}_{c}+1.45\cdot {N}_{c}+1.84\cdot {T}_{c}.$$

Logistic regression based on species-level ecological classes derived from Ellenberg indicator values revealed significant effects of trophic status (*N*_c_) and temperature (*T*_c_) on invasion status. The probability of a species being classified as invasive increased significantly with increasing trophic class (1.45, *p* = 0.002, Table 1) and temperature class (1.84, *p* = 0.005, Table 1). Reaction class showed a negative but only marginally significant association with invasion status (− 0.74, *p* = 0.056, Table 1), suggesting a tendency for invasive species to be less frequently associated with alkaliphilous habitats. Moreover, it can be concluded that with an increase in the species’ trophic preferences by one class (e.g. from oligotrophic to mesotrophic species or from mesotrophic to eutrophic), the chance of classifying the species as an invasive species increased approximately 4.3 times, while with an increase in the temperature class by one level (e.g. from cold-loving to temperate species), the probability of classifying the species as invasive increased approximately 6.3 times (OR, Table 1).

**Table 1:** Parameter estimates for the logistic regression model of invasion status

Statistical measures	${\alpha}_{0}$	${R}_{c}$	${N}_{c}$	${T}_{c}$
estimation of ${\alpha}_{k}$	− 8.10*	− 0.74	1.45*	1.84*
SE	1.73	0.39	0.47	0.65
*p*-value	< 0.0001	0.0560	0.0021	0.0049
odds ratio (OR)	0.0003	0.4792	4.2832	6.3172

α_0_ means model intercept, *R*_*c*_*, N*_*c*_*, T*_*c*_ = species-level Ellenberg indicator classes for soil reaction, nitrogen and temperature, respectively (Zarzycki et al. 2002; Supplementary Table S6), *SE* = standard error, *OR* = odds ratio

^*^means statistical significance at the 0.05 level

**Fig. 6:**
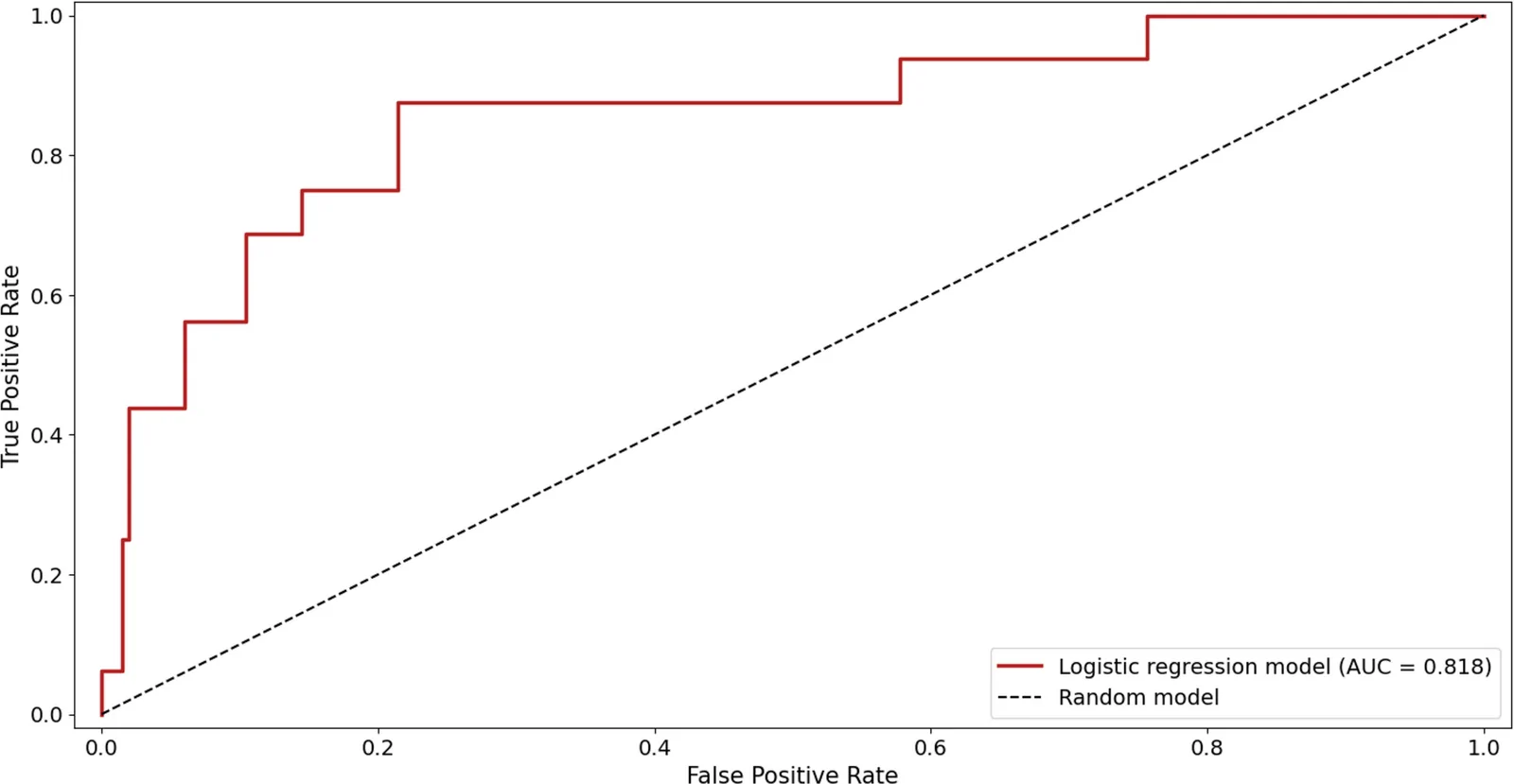
Receiver operating characteristic (ROC) curve for the logistic regression model relating invasion status to species-level Ellenberg indicator classes (*T*_c_, *R*_c_, *N*_c_). The model achieved an AUC of 0.818, indicating good discrimination between invasive and non-invasive species

Model fit statistics indicated that the exploratory logistic regression captured non-random structure in the data (AIC = 107.39, McFadden’s pseudo-*R*^2^ = 0.212). ROC analysis yielded an AUC of 0.818 (Fig. 6), indicating good discrimination between invasive and non-invasive observations within the studied dataset.

### Ecological status classification based on the Index of Biotic Integrity (IBI)

A composite Index of Biotic Integrity (IBI) was calculated to summarise multiple dimensions of vegetation condition, including floristic diversity, the proportion of alien and invasive taxa, Ellenberg indicator values for nitrogen and moisture, and vegetation cover structure. The total IBI score ranges from 0 to 30, with higher values indicating a greater abundance of natural riparian vegetation.

**Fig. 7:**
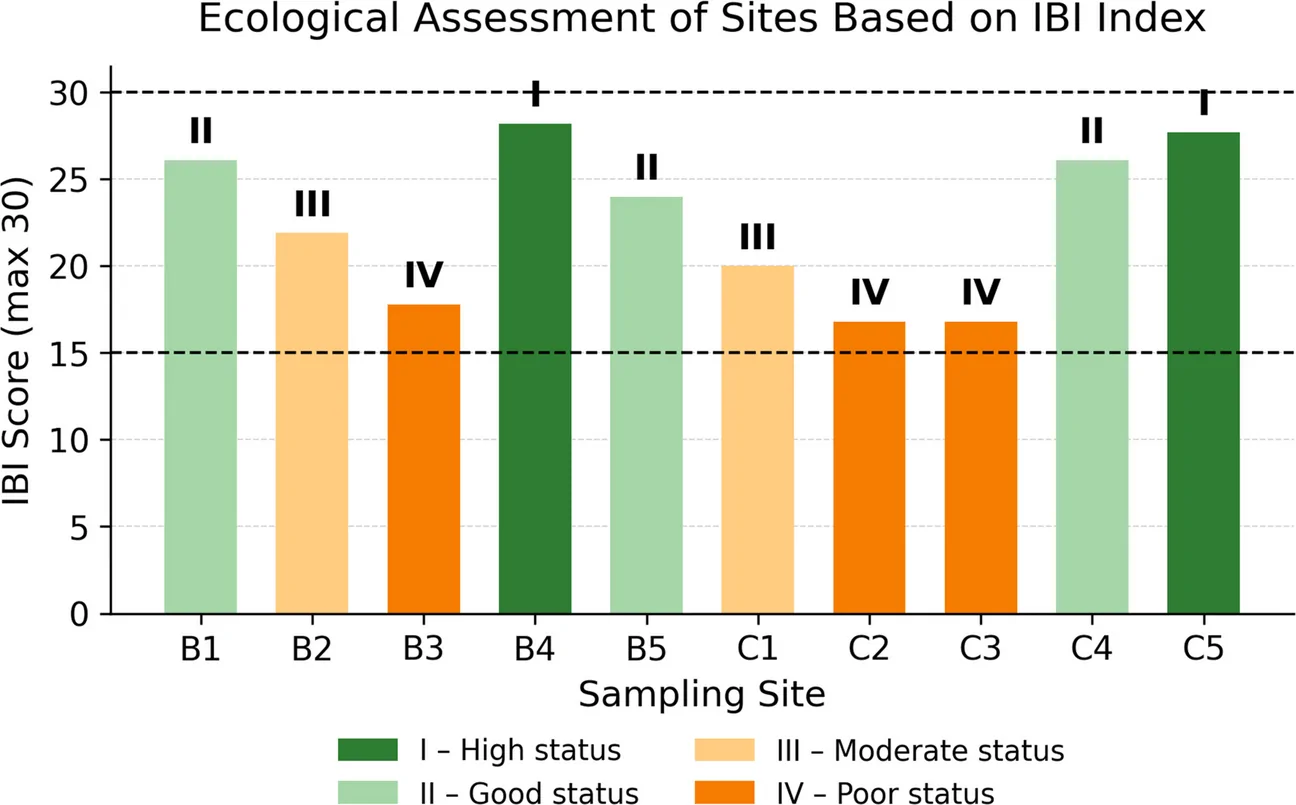
Site-level Index of Biotic Integrity (IBI) scores for the ten study sites. Bars represent total IBI scores (range 0–30), with classes used here as comparative categories within the study. I—very good, II—good, III—moderate, IV—poor, V—bad. The dashed lines correspond to the min and max values of the IBI index

Across the ten sites, IBI values ranged from 17 to 28 (Fig. 7). Higher IBI classes were recorded at B1, B4, B5, C4 and C5, whereas lower classes were recorded at B3, C2 and C3. These classes are interpreted comparatively within this study rather than as calibrated regulatory status categories. Moderate quality (IBI 19–22) occurred at B2 and C1, while poor quality (IBI 15–18) was recorded at B3, C2 and C3. The IBI indicated variation in ecological quality among the studied site units. Some site units, including B4, B5, C4 and C5, received higher IBI scores, whereas others, including B2, B3, C2 and C3, received lower scores. Because land-use categories were not independently replicated, these differences are interpreted as local site-level observations rather than evidence of land-use effects. Sites B1 and C1 occupied intermediate positions along the IBI gradient.

## Discussion

This study aimed to assess floristic diversity, vegetation structure and the occurrence of invasive alien species (IAS) in riparian ecosystems of two lowland river valleys. The clearest result was obtained from the species-level logistic regression, which showed that invasive taxa differed from non-invasive taxa in their Ellenberg-based ecological preference profiles. In particular, invasive species were more frequently associated with higher nitrogen and temperature, suggesting a stronger representation of eutrophic and thermophilous taxa within the invasive species pool. It should be noted that transect-level mixed-effects models did not reveal significant relationships between proportional IAS cover and weighted mean Ellenberg indicator values. Thus, there was no statistical basis for identifying a relationship between proportional IAS cover and the habitat conditions considered in this study.

Floristic diversity, community evenness and vegetation integrity varied among the studied site units, but these differences should be interpreted as local descriptive observations rather than as formal evidence of land-use effects. This distinction is particularly important because land-use categories were weakly replicated and some local land-use contexts occurred only in one of the two studied rivers. Consequently, the observed variation in vegetation composition and IBI values cannot be attributed unambiguously to land-use category alone.

### Ecological preference profiles of invasive and non-invasive species

The species-level analysis showed that invasive and non-invasive taxa differed in their Ellenberg-based ecological preference profiles. Among the analysed indicator classes, nitrogen and temperature showed the clearest associations with invasion status. Species belonging to higher nitrogen indicator classes were more likely to be classified as invasive, suggesting that the invasive species were represented by taxa with eutrophic ecological preferences. This is consistent with previous studies showing that nutrient enrichment can favour fast-growing and disturbance-tolerant alien plants (Vilà and Weiner 2004; Vilà et al. 2011). Temperature indicator class was also positively associated with invasion status. This indicates that invasive taxa recorded in the studied river valleys were more frequently represented among species with higher temperature preferences. Such thermophilous tendencies may be relevant in riparian corridors, where disturbance, dispersal opportunities and changing climatic conditions can facilitate the establishment and spread of alien plant species.

Reaction class showed a negative but only marginally significant association with invasion status. This result suggests a possible tendency for invasive taxa to be less frequently represented among species associated with higher reaction classes, although this relationship should be interpreted cautiously because it did not reach conventional statistical significance. Therefore, reaction should be treated as a tentative ecological signal rather than as a robust predictor of invasion status. Light and moisture indicator classes did not show independent associations with invasion status in the final model. Their role may therefore be less direct or may overlap with other aspects of species ecology and vegetation structure. To sum up, the results indicate that invasive taxa in the studied river valleys were characterised primarily by higher nitrogen and temperature Ellenberg indicator classes.

### Floristic diversity, land use and invasion patterns in river valleys

Disturbance and habitat simplification have been widely linked to reduced species richness and evenness in river systems elsewhere in Europe (Aguiar et al. 2001; Jurasinski et al. 2009; Aguiar and Ferreira 2013; Brazuelo Núñez et al. 2025). As a site-level observation, however, the highest Shannon diversity (*H*′) in this study was recorded at site C2, associated with hydrotechnical infrastructure, whereas comparatively lower values were recorded at B5 (meadow) and C1 (agricultural land). Because land-use categories were not sufficiently replicated in this study design, these observations cannot be used to test for land-use effects on floristic diversity and are reported here only as site-specific descriptive patterns.

As a further site-level observation, relatively high Shannon diversity was also recorded at the allotment garden site (B1). By contrast, comparatively lower Shannon diversity was recorded at the two meadow sites (B5, C5), both characterised by relatively high nitrogen indicator values. This design does not allow the underlying mechanisms behind these patterns to be tested. Low Shannon diversity was not consistently associated with the same dominance structure. Sites with similar *H*′ values differed markedly in Simpson Index values, indicating that reduced diversity may result either from strong dominance by a few species or from species-poor but relatively even assemblages. This pattern highlights the value of combining complementary diversity metrics when interpreting floristic homogenisation and community structure.

Invasive taxa were recorded across multiple habitat contexts. Herbaceous invaders were more frequently observed in disturbed and nutrient-enriched sites, whereas woody alien taxa were more often associated with built-up and infrastructure-related habitats. These patterns suggest that different groups of invasive species are associated with different habitat conditions within riparian systems.

IAS occurrence varied across the studied sites, but this variation did not correspond to a statistically supported habitat pattern: transect-level models did not detect a significant relationship between proportional IAS cover and weighted mean Ellenberg indicator values. As a site-level observation, the two meadow sites (B5, C5) had comparatively higher nitrogen indicator values alongside relatively high IAS occurrence; however, this association was not statistically tested and should not be interpreted as evidence of a land-use or habitat effect.

### Invasive species and biodiversity in riparian systems

The clearest evidence from this study is the species-level association between invasion status and ecological preference reported in the “Ecological preference profiles of invasive and non-invasive species” section; no significant relationship between IAS cover and habitat conditions was detected at the transect level. Anecdotally, higher IAS occurrence at some sites coincided with reduced Shannon diversity, lower evenness and increased dominance, a pattern broadly consistent with previous reports linking invasive species to shifts in community composition and dominance (McKinney and Lockwood 1999; Olden and Rooney 2006). This pattern is also consistent with Elton’s (1958) hypothesis that structurally complex, more diverse plant communities show greater resistance to invasion by alien species.

The composition of IAS varied among individual site-level units in both river valleys. Along the Biala Lada, the recorded IAS included mainly herbaceous ruderal taxa such as *Solidago gigantea*, *Erigeron canadensis* and *Echinochloa crus-galli*. Along the Czarna Lada, the IAS pool included a greater representation of woody alien taxa, including *Robinia pseudoacacia*, *Prunus serotina* and *Rhus typhina*. These observations are interpreted descriptively as local differences in species composition rather than as formal evidence of river or land-use-specific effects.

The occurrence of IAS within the studied sites should therefore be interpreted in relation to local variation in vegetation composition and community structure, rather than as evidence of generalised habitat effects. This distinction is important because land-use types were not independently replicated. Consequently, observed differences in diversity metrics, dominance structure and IAS composition are treated as local descriptive patterns within the studied river valleys.

### Vegetation-based Index of Biotic Integrity as a synthetic measure of riparian vegetation integrity

Beyond individual floristic metrics, the vegetation-based Index of Biotic Integrity (IBI) provided an integrated description of variation in vegetation structure and species composition among the studied riparian site-level units. By combining diversity indices, the proportion of alien and invasive taxa, selected Ellenberg indicator values (notably nitrogen and temperature moisture), and vegetation structure components, the IBI summarised multiple aspects of vegetation integrity within the analysed river valleys.

IBI scores ranged from 17 to 28 (out of 30), indicating variation among sites in vegetation structure, species composition and the relative contribution of alien and invasive taxa. Higher values were observed at sites with more structurally complex vegetation and a higher representation of native species, whereas lower values occurred at sites characterised by simplified vegetation and a greater contribution of alien taxa. Intermediate values reflected mixed structural and compositional characteristics. Because land-use categories were not independently replicated, these differences are interpreted as site-level observations rather than as formal evidence of land-use effects.

The IBI therefore served as a synthetic descriptive metric for summarising local variation in vegetation integrity across the studied site-level units. Similar multimetric approaches are commonly used in ecological assessment frameworks because they integrate several complementary aspects of biological condition rather than relying on single diversity measures alone (Karr and Chu 1999). In the present study, the inclusion of alien and invasive species metrics allowed the index to capture aspects of community composition that are particularly relevant in human-modified riparian environments. As noted in previous studies, diversity metrics alone may not fully reflect vegetation structure and composition when species origin is not considered (Pauchard et al. 2018).

From a management perspective, the IBI may provide a useful screening tool for identifying site units where vegetation structure and species composition indicate reduced ecological integrity. In this context, vegetation-based indicators such as Ellenberg values and IBI scores may complement existing assessment approaches applied in European policy frameworks, including EU Regulation 1143/2014. However, given the pilot scale of this study and the limited replication of land-use categories, the IBI is interpreted here as a comparative and descriptive tool for summarising variation among the studied site units, rather than as a basis for generalised inference about land-use effects or formal regulatory ecological classification.

## Conclusions

This study of two lowland river valleys in eastern Poland provides an exploratory assessment of riparian vegetation composition, floristic diversity, and the occurrence of invasive alien plant species (IAS). The results suggest that invasive and non-invasive taxa occupy different ecological niches, as reflected by their literature-based Ellenberg indicator classes. Invasive taxa were associated with higher nitrogen and temperature values, indicating a preference for eutrophic and warmer habitats.

At the site-level, variation in floristic diversity, dominance structure and IAS occurrence was observed among the studied riparian units. In several site units, higher IAS occurrence coincided with reduced community evenness and greater dominance of ruderal or alien taxa. These observations suggest local shifts in vegetation structure, but they should be interpreted descriptively because land-use categories were not independently replicated and some land-use contexts occurred only in one of the two studied rivers.

From a management perspective, riparian corridors may function both as reservoirs of native biodiversity and as pathways for the spread of alien plants. Vegetation-based indicators, particularly Ellenberg indicator values and the vegetation-based Index of Biotic Integrity (IBI), were useful for summarising variation in vegetation structure and species composition and highlighting differences among sites. These approaches may complement existing vegetation monitoring frameworks applied within European policy contexts, including EU Regulation 1143/2014 (European Parliament and Council 2014).

The study highlights that Ellenberg indicator values and vegetation integrity metrics can provide complementary information on invasion-related ecological patterns in riparian systems. The findings should be interpreted within the local context of the studied river valleys and used as a basis for further studies with stronger replication of land-use categories or continuous landscape-scale predictors.

## Supplementary Information

Below is the link to the electronic supplementary material.

ESM 1 (DOCX 413 KB)

## Data Availability

The data that support the findings of this study are available on request from the corresponding author.
