# Supplementary material for: Invasive alien plants in riparian ecosystems: a study from two lowland rivers in Eastern Poland
Source: Environ Sci Pollut Res Int. 2026 Oct 7;33(35):16969–82. doi: 10.1007/s11356-026-38279-6 (PMC13683481; doi:10.1007/s11356-026-38279-6)
Supplement: Supplementary file 1 [file 11356_2026_38279_MOESM1_ESM.docx]

**Figure S1**. A schematic representation of the spatial arrangement of transects (T1-T5) and vegetation plots within a study site

**Table S1**. River parameters at the analysed sections

| study point | **Biała Łada** | | | | | **Czarna Łada** | | | | |
| --- | --- | --- | --- | --- | --- | --- | --- | --- | --- | --- |
| Land-use category | B1 Allotment gardens | B2 Hydrote -chnical infrastructure | B3 Residential areas | B4 Tree plantations | B5 Meadows | C1 Agricul -tural land | C2 Hydrote -chnical infrastructure | C3 Residential areas | C4 Tree plantations | C5 Meadows |
| N/E | 50°54’53”, 22°51’50” | 50°54’59”, 22°51’68” | 50°53’20”, 22°51’16” | 50°54’42”, 22°51’53” | 50°53’56”, 22°51’26” | 50°51’49”, 22°50’73” | 50°52’08”, 22°51’14’ | 50°52’20”, 22°51’27” | 50°52’05”, 22°52’33” | 50°52’08”, 22°52’43” |
| river channel width (m) | 3.7 | 3.8 | 4.4 | 3.9 | 3.5 | 3.2 | 3.1 | 2.9 | 3.7 | 3.6 |
| depth (m) | 0.45 | 0.55 | 0.6 | 0.5 | 0.55 | 0.7 | 0.4 | 0.8 | 1.15 | 1.1 |
| flow velocity (m/s) | 1.25 | 1 | 0.71 | 1 | 0.83 | 0.71 | 1 | 0,6 | 0.5 | 0.5 |
| substrate type | Sand /gravel | Sand / gravel | Sand | Sand / gravel | Sand | Silt | Sand / Silt / Stones | Silt / Stones | Sand / Silt | Sand / Silt |
| bank slope (‰) | 397 | 459.4 | 333.3 | 566.6 | 542.8 | 933.3 | 545.45 | 714.3 | 600 | 555.6 |
| Distance to development (m) | 20 | 22 | 48 | 20 | 80 | 30 | 50 | 5 | 60 | 160 |
| Modifications | Mowing of riverbanks | River channel regulated within the bridge area / Mowing of riverbanks | Numerous stormwater outfalls | Regular mowing of riverbanks | Regular mowing of riverbanks | Regular mowing of riverbanks | Channel regulation within the bridge area / Riverbanks reinforced with stones near the bridge / Flood embankments | Left bank regulated, reinforced with stone riprap | No modifications observed | No modifications observed |

**Table S2.** Index of Biotic Integrity (IBI) Metric**.** Each metric was assigned a score on a scale from 1 to 5 based on reference conditions

| **Metric** | **Scale** | **Score** | **Description** |
| --- | --- | --- | --- |
| Plant species diversity (Shannon–Wiener index) | < 1.0 | 1 | Very low diversity – heavily degraded environment |
|  | 1.0 – 1.5 | 2 | Low diversity – significant ecological disturbance |
|  | 1.51 – 2.5 | 3 | Moderate diversity – moderate anthropogenic impact |
|  | 2.51 – 3.5 | 4 | High diversity – good ecological conditions |
|  | >3.50 | 5 | Very high diversity – natural or near-natural environment |
| Presence of invasive species | >40% | 1 | Very high pressure |
|  | 21 – 40% | 2 | High pressure |
|  | 11 – 20% | 3 | Moderate pressure |
|  | 1-10% | 4 | Low presence of invasive species |
|  | 0% | 5 | No invasive species present |
| Proportion of non-native species | >30% | 1 | Dominance of non-native species – strong anthropogenic pressure |
|  | 21 – 30% | 2 | Significant presence of non-native species |
|  | 11 – 20% | 3 | Moderate presence of non-native species |
|  | 1 – 10% | 4 | Low impact of non-native species |
|  | 0% | 5 | No non-native species – natural flora composition |
| River valley modifications |  | 5 | No modifications, natural banks and riverbed |
|  |  | 4 | Minimal modifications |
|  |  | 3 | Localized modifications |
|  |  | 2 | Significant localized modifications |
|  |  | 1 | Severe modifications of the valley/riverbed |
| Land use |  | 5 | Natural / near-natural |
|  |  | 4 | Extensive agricultural use |
|  |  | 3 | Intensive agriculture |
|  |  | 2 | Scattered housing / rural settlements |
|  |  | 1 | Urban / industrial development / infrastructure |
| Average value of the N index for the site (weighted or arithmetic mean) | ≤ 3.0 | 5 | Very poor, natural habitat; No signs of eutrophication |
|  | 3.1 – 4.0 | 4 | Moderately rich habitat; Slight impact of eutrophication |
|  | 4.1 – 5.5 | 3 | Neutral or slightly enriched environment; Moderate impact |
|  | 5.6 – 6.5 | 2 | Nitrogen-enriched, anthropogenic influence; Significant nitrogen pressure |
|  | >6.5 | 1 | Strong eutrophication, habitat degradation; Dominance of nitrophilous species |
| Proportion of synanthropic species | >40% | 1 | Dominance of ruderal species – severe environmental degradation |
|  | 21 – 40% | 2 | Strong anthropogenic pressure |
|  | 11 – 20% | 3 | Moderate impact |
|  | 1-10% | 4 | Low human impact |
|  | 0% | 5 | No anthropogenic impact |
| Average value of the F index for the site (weighted or arithmetic mean) | ≥ 7.5 | 5 | Typical aquatic, wet, natural habitat; Dominance of aquatic and marsh species |
|  | 6.5 – 7.4 | 4 | Moist habitat, good hydrological condition; High proportion of moisture-loving plants |
|  | 5.5 – 6.4 | 3 | Moderately moist, potential modifications; Moderate presence of moisture-loving plants |
|  | 4.0 – 5.4 | 2 | Lowered water level, drying out |
|  | < 4.0 | 1 | Severe drying, aquatic habitat degradation; Dominance of drought-tolerant species |

**Table S3**. Ecological status classification based on the IBI

| **IBI score range** | **Ecological status class** | **Description** |
| --- | --- | --- |
| **27–30** | I - very good | Near-natural condition, with no signs of pressure |
| **23–26** | II – good | Minor impacts, ecosystem in balance |
| **19–22** | III – moderate | Visible anthropogenic impact, partial modification |
| **15–18** | IV – poor | Significant habitat and/or flora modification |
| **< 15** | V – very poor | Severe environmental degradation, loss of natural features |

**Table S4.** List of invasive plant species recorded across study sites along the Biała Łada (B1–B5) and Czarna Łada (C1–C5) rivers, with the number of observations per site. The table includes all invasive taxa identified based on national and international databases and literature. Species are grouped by river and site, reflecting their frequency of occurrence in floristic plots (SD - standard deviation)*.*

| river | site | species | mean±SD [%] |
| --- | --- | --- | --- |
| B | 1 | *Mahonia aqyifolium* | 0.1±0.2 |
|  |  | *Helianthus tuberosus* | 0.1±0.2 |
|  |  | *Lupinus polyphyllus* | 0.1±0.2 |
|  |  | *Solidago gigantea* | 3.6±6.5 |
|  | 2 | *Erigeron annuus* | 3.3±6.5 |
|  |  | *Solidago gigantea* | 0.6±1.3 |
|  | 3 | *Juglans regia* | 0.1±0.2 |
|  |  | *Echinochloa crus-galli* | 3.6±6.5 |
|  |  | *Setaria viridis* | 0.6±1.3 |
|  |  | *Erigeron canadensis* | 0.7±1.3 |
|  |  | *Erigeron annuus* | 0.1±0.2 |
|  | 4 | *Juglans regia* | 0.1±0.2 |
|  |  | *Solidago gigantea* | 0.6±1.3 |
|  | 5 | *Prunus serotina* | 3.1±6.7 |
| C | 1 | *Solidago gigantea* | 6.0±8.2 |
|  |  | *Erigeron annuus* | 0.8±1.3 |
|  | 2 | *Robinia pseudoacacia* | 3.1±6.7 |
|  |  | *Acer negundo* | 0.1±0.2 |
|  |  | *Erigeron annuus* | 1.2±1.6 |
|  |  | *Vicia sativa* | 4.2±6.2 |
|  |  | *Solidago gigantea* | 10.5±14.5 |
|  | 3 | *Robinia pseudoacacia* | 0.6±1.3 |
|  |  | *Juglans regia* | 0.1±0.2 |
|  |  | *Solidago gigantea* | 0.6±1.3 |
|  |  | *Rhus typhina* | 0.1±0.2 |
|  |  | *Erigeron annuus* | 0.6±1.3 |
|  | 4 | *Prunus serotina* | 9.0±8.2 |
|  |  | *Solidago gigantea* | 6.0±8.2 |
|  |  | *Ssolidago canadensis* | 3.0±6.7 |
|  | 5 | *Prunus serotina* | 0.6±1.3 |
|  |  | *Solidago gigantea* | 9.8±15 |

**Table S5.** Matrix of PC1-PC3 factor loadings calculated based on climatic indicators and structural habitat features

| Indicator | PC1 | PC2 | PC3 |
| --- | --- | --- | --- |
| L | -0.90 | -0.19 | -0.37 |
| T | -0.47 | -0.27 | 0.64 |
| F | 0.63 | 0.63 | -0.32 |
| R | -0.47 | 0.70 | 0.20 |
| N | -0.20 | 0.95 | 0.11 |
| TC% | 0.88 | 0.02 | 0.57 |
| SC% | 0.16 | -0.89 | -0.36 |
| HV% | -0.91 | 0.35 | -0.39 |
| WR | 0.99 | 0.09 | -0.11 |
| BS | -0.75 | -0.24 | 0.53 |
| Eigenvalue | 4.88 | 2.89 | 1.61 |
| Percentage of variance explained [%] | 43.94 | 26.04 | 14.45 |
| Ellenberg indicator values (L, T, F, R, N), tree (TC%), shrub (SC%) and herbaceous (HV%) cover and channel morphology variables (riverbed width (WR) and bank slope (BS)). | | | |

**Table S6.** Ranges of ecological classes according to Ellenberg indicator values

| **Indicator** | **Numerical range** | **class of indicator** | **Ecological class** |
| --- | --- | --- | --- |
| **L_c_ (light)** | 1–3 | 1 | shade-affinity |
|  | 4–6 | 2 | moderate light requirements |
|  | 7–9 | 3 | light-affinity |
| **F_c_ (moisture)** | 1–3 | 1 | drought-tolerant |
|  | 4–6 | 2 | mesophilous |
|  | 7–9 | 3 | moisture-affinity |
|  | 10–12 | 4 | hydrophilous (wetlands, reed beds) |
| **R_c_ (raction)** | 1–3 | 1 | acidophilous |
|  | 4–6 | 2 | neutral |
|  | 7–9 | 3 | alkaliphilous |
| **N_c_ (trophy)** | 1–3 | 1 | oligotrophic (poor) |
|  | 4–6 | 2 | mesotrophic (moderately nutrient-rich) |
|  | 7–9 | 3 | eutrophic (rich in nutrients) |
| **T_c_ (temperature)** | 1–3 | 1 | cold-tolerant |
|  | 4–6 | 2 | moderately tolerant |
|  | 7–9 | 3 | heat-tolerant |
| adapted from: Zarzycki K, Trzcinska-Tacik H, Rozanski W, Szalag Z, Wolek J, Korzeniak U (2002) Ecological indicator values of vascular plants of vascular plants of Poland. Biodiversity of Poland (Poland). W. Szafer Institute of Botany, PAN. | | | |
